# Letter from the editor

**DOI:** 10.1007/s00775-022-01951-6

**Published:** 2022-08-10

**Authors:** Nils Metzler-Nolte

**Affiliations:** Chair of Inorganic Chemistry I, Bioinorganic Chemistry, Faculty of Chemistry and Biochemistry, Ruhr University Bochum, Bochum, Germany

Dear JBIC Reader, Dear Colleague,

It is my pleasure to congratulate Professor Tim Storr from Simon Fraser University, Canada, who is the recipient of the 2021 SBIC Early Career Award!

Tim receives the award for his work in medicinal inorganic chemistry, particularly for his work to use metal complexes to target dysregulated metal ions and protein aggregation in diseases, and you can read about the highly collaborative work of the group in a minireview in this Issue of JBIC [1]. In addition, his group has produced a wonderful video to go with the article which explains their motivation and the background of the work [2]. It is a first for JBIC to publish additional material of such nature, and I am probably as excited as the authors to get this video out, and to hear your feedback on this new feature in JBIC!

Tim completed his Ph.D. in medicinal inorganic chemistry at the University of British Columbia with Prof. Chris Orvig, and then studied metalloenzyme mimics as a postdoctoral fellow with Prof. Dan Stack at Stanford, before beginning his independent career at Simon Fraser University, Canada, where he is now a professor of chemistry. He has made diverse contributions to the field of medicinal inorganic chemistry, including the use of multifunctional ligands and metal complexes to target dysregulated metal ions and protein aggregation in neurodegenerative diseases and cancer. Beyond medicinal inorganic chemistry, his fundamental research on redox-active ligands has allowed his team to turn on metal nitride reactivity based on peripheral electronics, and design tunable NIR-absorbing molecules.

**Figure:**
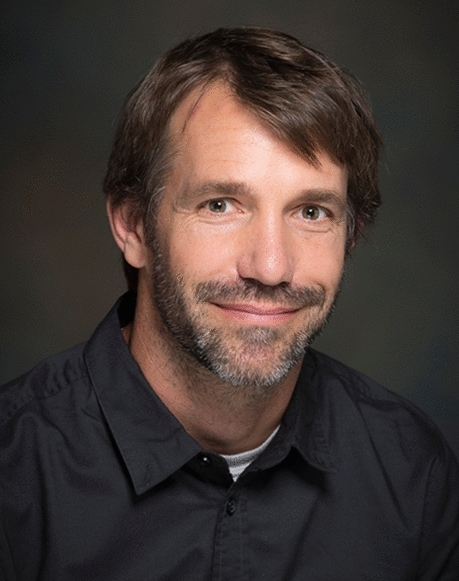
Prof. Tim Storr, Recipient of the 2021 SBIC Early Career Award

Tim is well known to the Bioinorganic Community as the Editor of a book entitled ‘Ligand Design in Medicinal Inorganic Chemistry,’ (published by Wiley in 2014) that many of us use as a source of information and especially a teaching resource. Tim also serves the bioinorganic community, amongst other roles, as an Associate Editor of the Encyclopedia of Inorganic and Bioinorganic Chemistry, and in 2019, he received the Strem Chemicals Award for Pure or Applied Inorganic Chemistry from the Canadian Society for Chemistry.

It has been a pleasure working with Tim and Jessica not only to get their minireview published but also to produce the additional material. Encouraged by the experience, we at JBIC shall be happy to feature more additional audio-visual material for the promotion of articles, public outreach, or community engagement in the future. If you have an idea for such material (it need not be just videos, also audio material, animated slides, cartoons, etc. would be highly welcome!) please get in touch with me directly, and together with our Publisher’s production and promotion team and our Society, SBIC, we can actually achieve significant outreach.

Therefore, this time, I close with more than a reading recommendation: Enjoy reading *and watching*!

Nils Metzler-Nolte

Editor-in-Chief, JBIC
